# Supplementary material for: Relationships between personality, emotional well-being, self-efficacy and weight management among adults with type 2 diabetes: Results from a cross-sectional survey
Source: PLoS One. 2023 Oct 30;18(10):e0292553. doi: 10.1371/journal.pone.0292553 (PMC10615271; doi:10.1371/journal.pone.0292553)
Supplement: S2 Table — (DOCX) [file pone.0292553.s003.docx]

## Supplemental Table S2. Regression model with cross-sectional explanatory variables for physical activity, reorganised utilising diabetes distress subscales to identify source of suppressor effect (N=205)

| *Model* | *1* | *2* | *3* | *4* | *5* | *6* | *7* | *8* | *9* |
| --- | --- | --- | --- | --- | --- | --- | --- | --- | --- |
| *Variables* | | | | | | | | | |
| Age | -0.02 | -0.02 | -0.03 | -0.05 | -0.04 | -0.04 | -0.04 | -0.09 | -0.09 |
| Female gender | -0.20** | -0.19** | -0.19* | -0.17 | -0.17* | -0.12 | -0.11 | -0.10 | -0.16* |
| Diabetes duration | -0.03 | -0.03 | -0.02 | -0.02 | -0.02 | -0.03 | -0.03 | -0.05 | -0.07 |
| Diabetes comorbidities | -0.14 | -0.14 | -0.13 | -0.13 | -0.13 | -0.07 | -0.08 | -0.06 | -0.06 |
| Physician related distress |  | -0.01 | 0.01 | 0.01 | -0.01 | 0.05 | 0.03 | 0.02 | 0.03 |
| Interpersonal distress |  |  | -0.07 | -0.03 | -0.09 | -0.07 | -0.07 | 0.02 | 0.04 |
| Regimen distress |  |  |  | -0.09 | -0.17 | 0.17 | 0.16 | 0.16 | 0.13 |
| Emotional burden |  |  |  |  | 0.16 | 0.11 | 0.14 | 0.16 | 0.13 |
| Diabetes self-efficacy |  |  |  |  |  | 0.53** | 0.50** | 0.37** | 0.32** |
| General self-efficacy |  |  |  |  |  |  | 0.08 | -0.04 | -0.06 |
| General well-being |  |  |  |  |  |  |  | 0.40** | 0.45** |
| Honesty-Humility |  |  |  |  |  |  |  |  | 0.05 |
| Emotionality |  |  |  |  |  |  |  |  | 0.15* |
| Extraversion |  |  |  |  |  |  |  |  | 0.00 |
| Agreeableness |  |  |  |  |  |  |  |  | -0.06 |
| Conscientiousness |  |  |  |  |  |  |  |  | 0.11 |
| Openness |  |  |  |  |  |  |  |  | 0.15* |
| Adjusted R^2^ | .04* | .03 | .03 | .03 | .04 | .19 | .20 | .27 | .30 |
| Adjusted R^2^ Change |  | 0 | 0 | 0 | .01 | .15** | .01 | .07** | .03* |

* p<0.05 ** p<0.01
