# Supplementary material for: Relationships between personality, emotional well-being, self-efficacy and weight management among adults with type 2 diabetes: Results from a cross-sectional survey
Source: PLoS One. 2023 Oct 30;18(10):e0292553. doi: 10.1371/journal.pone.0292553 (PMC10615271; doi:10.1371/journal.pone.0292553)
Supplement: S3 Table — (DOCX) [file pone.0292553.s004.docx]

## Supplemental Table S3. Correlations between personality facets and weight management, general well-being and self-efficacy and diabetes-specific distress and self-efficacy *(N=270^)*

| Facet | Physical activity | Healthy diet | BMI | General emotional well-being | General self-efficacy | Diabetes distress | Diabetes  self-efficacy |
| --- | --- | --- | --- | --- | --- | --- | --- |
| Honesty-Humility (H) |  |  |  |  |  |  |  |
| H1. Sincerity | -.03 | .04 | .09 | -.15* | -.03 | .03 | .01 |
| H2. Fairness | .08 | .24** | -.02 | -.01 | -.09 | -.08 | .13* |
| H3. Greed-Avoidance | 0 | .11 | .11 | -.04 | -.07 | .03 | -.06 |
| H4. Modesty | -.01 | .12 | .11 | -.10 | -.22** | .16* | -.12 |
| Emotionality (E) |  |  |  |  |  |  |  |
| E1. Fearfulness | -.23** | -.08 | .08 | -.29** | -.41** | .18** | -.18** |
| E2. Anxiety | -.10 | -.16** | .21** | -.54** | -.43** | .35** | -.29** |
| E3. Dependence | .18** | .09 | .09 | -.01 | -.20** | .12 | -.05 |
| E4. Sentimentality | 0 | .05 | .18** | -.12* | -.25** | .18** | -.11 |
| Extraversion (X) |  |  |  |  |  |  |  |
| X1. Social Self-Esteem | .29** | .23** | -.25** | .71** | .55** | -.47** | .44** |
| X2. Social Boldness | .15* | .12 | -.10 | .34** | .41** | -.20** | .22** |
| X3. Sociability | .13 | .17** | -.06 | .36** | .28** | -.14* | .21** |
| X4. Liveliness | .32** | .24** | -.26** | .73** | .57** | -.40** | .45** |
| Agreeableness (A) |  |  |  |  |  |  |  |
| A1. Forgiveness | .03 | .13* | -.03 | .25** | .10 | -.16** | .12 |
| A2. Gentleness | .01 | .10 | .01 | .16** | .05 | -.12 | .15* |
| A3. Flexibility | .07 | .08 | -.03 | .15* | .15* | -.11 | .13* |
| A4. Patience | .06 | .10 | -.13* | .29** | .20** | -.20** | .18** |
| Conscientiousness (C) |  |  |  |  |  |  |  |
| C1. Organisation | .14* | .05 | -.04 | .17** | .26** | -.10 | .17** |
| C2. Diligence | .30** | .18** | -.13* | .34** | .46** | -.26** | .30** |
| C3. Perfectionism | -.02 | -.06 | .05 | -.13* | .06 | -.01 | -.01 |
| C4. Prudence | .08 | .07 | -.15* | .14* | .27** | -.15* | .12 |
| Openness (O) |  |  |  |  |  |  |  |
| O1. Aesthetic Appreciation | .13 | .05 | .01 | -.04 | .07 | .10 | .02 |
| O2. Inquisitiveness | .23** | .09 | -.23** | .19** | .20** | -.09 | .21** |
| O3. Creativity | .05 | -.06 | -.02 | .09 | .22** | -.02 | .10 |
| O4. Unconventionality | .18* | .04 | -.10 | .03 | .15* | .10 | .09 |
| Interstitial facet (I) |  |  |  |  |  |  |  |
| I. Altruism | .03 | .19** | .03 | .08 | -.06 | .03 | .01 |

* p<0.05 ** p<0.01

^Diabetes duration n=266, General wellbeing n=260, General self-efficacy n=260, Diabetes distress n=261, Diabetes self-efficacy n=262, Physical activity n=205, Healthy diet n=264 (due to pairwise analyses)
