# Supplementary material for: Relationships between personality, emotional well-being, self-efficacy and weight management among adults with type 2 diabetes: Results from a cross-sectional survey
Source: PLoS One. 2023 Oct 30;18(10):e0292553. doi: 10.1371/journal.pone.0292553 (PMC10615271; doi:10.1371/journal.pone.0292553)
Supplement: S1 File — (DOCX) [file pone.0292553.s005.docx]

Supplemental Results SR1. Description of facet-level correlations

## Facet-level correlates of personality: demographic, clinical, and psychosocial characteristics

Facet-level analysis (Supplemental Table S3) shows that the association between diabetes distress and emotionality was influenced most strongly by the facet anxiety (*r* = .35), and to extraversion by facets social self-esteem (*r* = -.47) and liveliness (*r* = -.40). The relationship between diabetes self-efficacy and conscientiousness and was influenced most strongly by the facet diligence (*r* = .30).

Facet-level correlates of weight management indicators

Facet-level analysis (Supplemental Table S3) shows that the relationships between extraversion and physical activity, healthy diet and BMI were influenced by two facets (social self-esteem: range *r* = -.25 – .29; liveliness: range *r* = -.26 – .32). The relationships between conscientiousness and physical activity and healthy diet were influenced most clearly by the facet diligence (*r* = .30 and .18 respectively). The associations between emotionality and physical activity, healthy diet and BMI differed, with facets fearfulness and dependence influencing the relationship with physical activity (*r* = -.23 and .18 respectively), anxiety influencing the relationship with healthy eating (*r* = -.16), and anxiety and sentimentality influencing the BMI relationship (*r* = .21 and .18 respectively). The relationship between openness and physical activity was influenced by the facet inquisitiveness (*r* = .23). Inquisitiveness also had a negative association with BMI (r = -.23). In addition, the relationship between honesty-humility and healthy eating was influenced by facet fairness (*r* = .24).

Facet-level involvement in weight management

Regarding the narrow traits measured, the positive relationship the facet dependency has with physical activity is supported by research demonstrating the positive effect dependency can have through supportive relationships (1). With diabetes distress and general emotional well-being likely accounting for the variance of the negative correlations of personality facets anxiety and fearfulness, the unique positive correlation of facet dependency may become amplified and influence the relationship between emotionality and physical activity in its explanation of variance. The significant correlation of physical activity with inquisitiveness, one of the facets of openness, may indicate that participants’ knowledge-seeking behavior about how to manage their diabetes through physical activity was responsible for openness’ contribution toward the explanation of variance in physical activity.

The utility of the honesty-humility domain in its explanation of variance in healthy diet appears to be influenced by the facet fairness, which has been postulated as one of the key differences between HEXACO and the Big Five in the conceptualisation of personality (2). A study on the perceived effectiveness of healthy eating interventions found that participants’ perception of how fair the intervention was, was the strongest predictor of its acceptance (3). This is an important implication in type 2 diabetes due to the many campaigns aimed at healthy eating. The conceptual underpinning of the fairness-acceptance relationship is that if a person’s dispositional perception of fairness aligns with a diabetes healthy eating campaign, they may be more likely to accept it and consume a healthier diet. Also, the facets social self-esteem, sociability and liveliness were significant positive correlates with healthy diet and may also influence extraversion’s relationship with that weight management indicator. However, given the sample size and number of comparisons made in this study, the facet level correlations do need to be interpreted with caution.

# References

1. Bornstein RF. Adaptive and maladaptive aspects of dependency: An integrative review. American Journal of Orthopsychiatry. 1994;64(4):622.

2. Lee K, Ashton MC. Psychometric Properties of the HEXACO Personality Inventory. Multivariate behavioral research. 2004;39(2):329-58.

3. Bos C, Lans IVD, Van Rijnsoever F, Van Trijp H. Consumer acceptance of population-level intervention strategies for healthy food choices: the role of perceived effectiveness and perceived fairness. Nutrients. 2015;7(9):7842-62.
