# Supplementary material for: Relationships between personality, emotional well-being, self-efficacy and weight management among adults with type 2 diabetes: Results from a cross-sectional survey
Source: PLoS One. 2023 Oct 30;18(10):e0292553. doi: 10.1371/journal.pone.0292553 (PMC10615271; doi:10.1371/journal.pone.0292553)

| **Coefficients Physical activity** | | | | | | | | |
| --- | --- | --- | --- | --- | --- | --- | --- | --- |
| Model | | Unstandardized Coefficients | | Standardized Coefficients | t | Sig. | Collinearity Statistics | |
|  |  | B | Std. Error | Beta |  |  | Tolerance | VIF |
| 1 | (Constant) | 3.835 | .527 |  | 7.279 | <.001 |  |  |
|  | Age | -.002 | .007 | -.020 | -.266 | .791 | .849 | 1.178 |
|  | Female gender | -.428 | .156 | -.196 | -2.742 | .007 | .939 | 1.065 |
|  | Diabetes specific comorbidities | -.128 | .064 | -.142 | -1.979 | .049 | .940 | 1.064 |
|  | Diabetes duration | -.004 | .012 | -.028 | -.379 | .705 | .909 | 1.100 |
| 2 | (Constant) | 3.209 | .652 |  | 4.922 | <.001 |  |  |
|  | Age | -.013 | .007 | -.144 | -2.012 | .046 | .782 | 1.278 |
|  | Female gender | -.250 | .146 | -.115 | -1.712 | .088 | .897 | 1.114 |
|  | Diabetes specific comorbidities | -.072 | .060 | -.080 | -1.211 | .227 | .917 | 1.091 |
|  | Diabetes duration | -.004 | .011 | -.026 | -.391 | .696 | .905 | 1.105 |
|  | General well-being | .081 | .014 | .447 | 5.678 | <.001 | .648 | 1.542 |
|  | General self-efficacy | -.006 | .015 | -.027 | -.364 | .716 | .726 | 1.378 |
| 3 | (Constant) | .798 | .795 |  | 1.004 | .317 |  |  |
|  | Age | -.009 | .006 | -.096 | -1.374 | .171 | .745 | 1.343 |
|  | Female gender | -.228 | .142 | -.105 | -1.603 | .111 | .847 | 1.181 |
|  | Diabetes specific comorbidities | -.057 | .057 | -.063 | -.994 | .321 | .889 | 1.124 |
|  | Diabetes duration | -.008 | .010 | -.052 | -.816 | .416 | .889 | 1.125 |
|  | General well-being | .073 | .015 | .404 | 4.806 | <.001 | .512 | 1.951 |
|  | General self-efficacy | -.011 | .015 | -.053 | -.738 | .462 | .708 | 1.413 |
|  | Diabetes distress | .020 | .006 | .287 | 3.586 | <.001 | .564 | 1.773 |
|  | Diabetes self-efficacy | .012 | .003 | .354 | 4.364 | <.001 | .550 | 1.817 |
| 4 | (Constant) | -1.727 | 1.210 |  | -1.426 | .155 |  |  |
|  | Age | -.008 | .006 | -.088 | -1.263 | .208 | .717 | 1.395 |
|  | Female gender | -.345 | .148 | -.158 | -2.329 | .021 | .753 | 1.328 |
|  | Diabetes specific comorbidities | -.057 | .057 | -.064 | -1.004 | .317 | .864 | 1.158 |
|  | Diabetes duration | -.011 | .010 | -.066 | -1.046 | .297 | .873 | 1.145 |
|  | General well-being | .081 | .017 | .449 | 4.797 | <.001 | .396 | 2.523 |
|  | General self-efficacy | -.013 | .017 | -.065 | -.777 | .438 | .491 | 2.035 |
|  | Diabetes distress | .018 | .006 | .253 | 3.176 | .002 | .547 | 1.827 |
|  | Diabetes self-efficacy | .010 | .003 | .303 | 3.738 | <.001 | .530 | 1.887 |
|  | Honesty-Humility | .119 | .147 | .052 | .808 | .420 | .855 | 1.169 |
|  | Emotionality | .299 | .142 | .152 | 2.103 | .037 | .662 | 1.510 |
|  | Extraversion | -.001 | .150 | .000 | -.005 | .996 | .414 | 2.415 |
|  | Agreeableness | -.133 | .133 | -.066 | -1.000 | .319 | .804 | 1.245 |
|  | Conscientiousness | .286 | .162 | .118 | 1.767 | .079 | .774 | 1.293 |
|  | Openness | .279 | .116 | .155 | 2.411 | .017 | .838 | 1.193 |
| Dependent Variable: Physical activity | | | | | | | | |


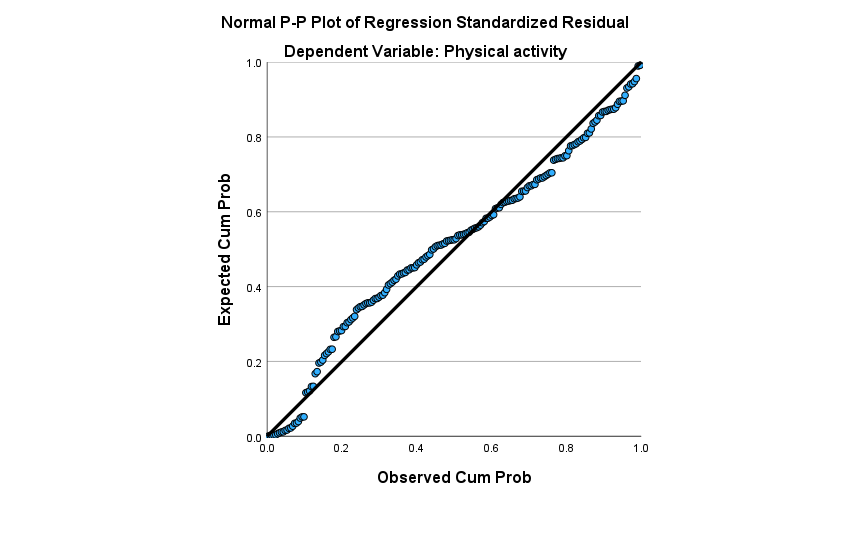


| **Coefficients Health diet** | | | | | | | | |  |
| --- | --- | --- | --- | --- | --- | --- | --- | --- | --- |
| Model | | Unstandardized Coefficients | | Standardized Coefficients | t | Sig. | Collinearity Statistics | |  |
|  |  | B | Std. Error | Beta |  |  | Tolerance | VIF |  |
| 1 | (Constant) | 59.227 | 5.136 |  | 11.532 | <.001 |  |  |  |
|  | Age | .267 | .068 | .258 | 3.912 | <.001 | .849 | 1.178 |  |
|  | Female gender | -.008 | 1.521 | .000 | -.006 | .996 | .939 | 1.065 |  |
|  | Diabetes specific comorbidities | -1.401 | .629 | -.140 | -2.229 | .027 | .940 | 1.064 |  |
|  | Diabetes duration | -.190 | .114 | -.106 | -1.670 | .096 | .909 | 1.100 |  |
| 2 | (Constant) | 58.615 | 6.838 |  | 8.572 | <.001 |  |  |  |
|  | Age | .206 | .070 | .199 | 2.933 | .004 | .782 | 1.278 |  |
|  | Female gender | .827 | 1.532 | .034 | .540 | .590 | .897 | 1.114 |  |
|  | Diabetes specific comorbidities | -1.096 | .627 | -.109 | -1.750 | .081 | .917 | 1.091 |  |
|  | Diabetes duration | -.193 | .112 | -.108 | -1.721 | .086 | .905 | 1.105 |  |
|  | General well-being | .454 | .150 | .226 | 3.034 | .003 | .648 | 1.542 |  |
|  | General self-efficacy | -.122 | .161 | -.053 | -.758 | .449 | .726 | 1.378 |  |
| 3 | (Constant) | 44.520 | 8.468 |  | 5.258 | <.001 |  |  |  |
|  | Age | .201 | .069 | .194 | 2.904 | .004 | .745 | 1.343 |  |
|  | Female gender | 2.159 | 1.516 | .089 | 1.424 | .156 | .847 | 1.181 |  |
|  | Diabetes specific comorbidities | -.655 | .612 | -.065 | -1.070 | .286 | .889 | 1.124 |  |
|  | Diabetes duration | -.187 | .109 | -.105 | -1.713 | .088 | .889 | 1.125 |  |
|  | General well-being | .159 | .162 | .079 | .980 | .328 | .512 | 1.951 |  |
|  | General self-efficacy | -.233 | .157 | -.102 | -1.488 | .138 | .708 | 1.413 |  |
|  | Diabetes distress | .013 | .061 | .016 | .207 | .836 | .564 | 1.773 |  |
|  | Diabetes self-efficacy | .129 | .029 | .349 | 4.499 | <.001 | .550 | 1.817 |  |
| 4 | (Constant) | 19.619 | 12.899 |  | 1.521 | .130 |  |  |  |
|  | Age | .195 | .069 | .189 | 2.827 | .005 | .717 | 1.395 |  |
|  | Female gender | .724 | 1.577 | .030 | .459 | .646 | .753 | 1.328 |  |
|  | Diabetes specific comorbidities | -.533 | .609 | -.053 | -.875 | .383 | .864 | 1.158 |  |
|  | Diabetes duration | -.198 | .108 | -.111 | -1.840 | .067 | .873 | 1.145 |  |
|  | General well-being | .011 | .180 | .005 | .059 | .953 | .396 | 2.523 |  |
|  | General self-efficacy | -.261 | .184 | -.114 | -1.416 | .158 | .491 | 2.035 |  |
|  | Diabetes distress | .023 | .060 | .030 | .388 | .698 | .547 | 1.827 |  |
|  | Diabetes self-efficacy | .122 | .029 | .330 | 4.247 | <.001 | .530 | 1.887 |  |
|  | Honesty-Humility | 5.013 | 1.562 | .196 | 3.209 | .002 | .855 | 1.169 |  |
|  | Emotionality | .921 | 1.514 | .042 | .608 | .544 | .662 | 1.510 |  |
|  | Extraversion | 3.377 | 1.601 | .185 | 2.109 | .036 | .414 | 2.415 |  |
|  | Agreeableness | -.003 | 1.421 | .000 | -.002 | .998 | .804 | 1.245 |  |
|  | Conscientiousness | .355 | 1.723 | .013 | .206 | .837 | .774 | 1.293 |  |
|  | Openness | -.856 | 1.235 | -.043 | -.693 | .489 | .838 | 1.193 |  |
| Dependent Variable: Healthy diet  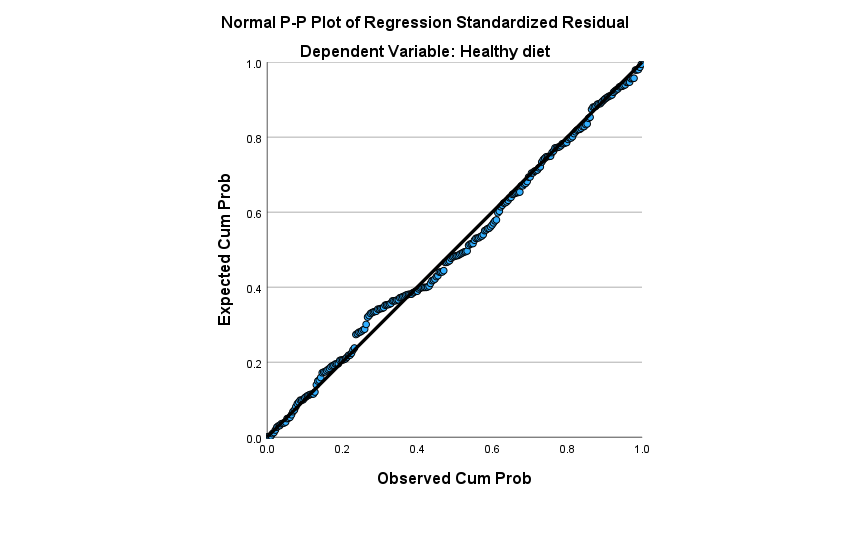 | | | | | | | | | |

| **Coefficients BMI** | | | | | | | | |
| --- | --- | --- | --- | --- | --- | --- | --- | --- |
| Model | | Unstandardized Coefficients | | Standardized Coefficients | t | Sig. | Collinearity Statistics | |
|  |  | B | Std. Error | Beta |  |  | Tolerance | VIF |
| 1 | (Constant) | 32.010 | 2.867 |  | 11.166 | <.001 |  |  |
|  | Age | -.140 | .038 | -.224 | -3.676 | <.001 | .849 | 1.178 |
|  | Female gender | 4.524 | .849 | .309 | 5.329 | <.001 | .939 | 1.065 |
|  | Diabetes specific comorbidities | 1.471 | .351 | .243 | 4.192 | <.001 | .940 | 1.064 |
|  | Diabetes duration | -.009 | .063 | -.008 | -.134 | .893 | .909 | 1.100 |
| 2 | (Constant) | 30.760 | 3.828 |  | 8.036 | <.001 |  |  |
|  | Age | -.109 | .039 | -.174 | -2.774 | .006 | .782 | 1.278 |
|  | Female gender | 4.164 | .857 | .284 | 4.856 | <.001 | .897 | 1.114 |
|  | Diabetes specific comorbidities | 1.310 | .351 | .216 | 3.736 | <.001 | .917 | 1.091 |
|  | Diabetes duration | -.004 | .063 | -.004 | -.067 | .947 | .905 | 1.105 |
|  | General well-being | -.243 | .084 | -.200 | -2.902 | .004 | .648 | 1.542 |
|  | General self-efficacy | .114 | .090 | .083 | 1.272 | .205 | .726 | 1.378 |
| 3 | (Constant) | 44.123 | 4.720 |  | 9.349 | <.001 |  |  |
|  | Age | -.128 | .038 | -.204 | -3.320 | .001 | .745 | 1.343 |
|  | Female gender | 3.811 | .845 | .260 | 4.509 | <.001 | .847 | 1.181 |
|  | Diabetes specific comorbidities | 1.158 | .341 | .191 | 3.396 | <.001 | .889 | 1.124 |
|  | Diabetes duration | .013 | .061 | .012 | .212 | .832 | .889 | 1.125 |
|  | General well-being | -.151 | .090 | -.124 | -1.674 | .095 | .512 | 1.951 |
|  | General self-efficacy | .159 | .087 | .115 | 1.824 | .069 | .708 | 1.413 |
|  | Diabetes distress | -.093 | .034 | -.194 | -2.740 | .007 | .564 | 1.773 |
|  | Diabetes self-efficacy | -.077 | .016 | -.344 | -4.806 | <.001 | .550 | 1.817 |
| 4 | (Constant) | 42.318 | 7.305 |  | 5.793 | <.001 |  |  |
|  | Age | -.116 | .039 | -.186 | -2.977 | .003 | .717 | 1.395 |
|  | Female gender | 3.698 | .893 | .252 | 4.140 | <.001 | .753 | 1.328 |
|  | Diabetes specific comorbidities | 1.144 | .345 | .189 | 3.316 | .001 | .864 | 1.158 |
|  | Diabetes duration | .013 | .061 | .012 | .205 | .837 | .873 | 1.145 |
|  | General well-being | -.149 | .102 | -.122 | -1.457 | .146 | .396 | 2.523 |
|  | General self-efficacy | .282 | .104 | .204 | 2.699 | .007 | .491 | 2.035 |
|  | Diabetes distress | -.087 | .034 | -.183 | -2.557 | .011 | .547 | 1.827 |
|  | Diabetes self-efficacy | -.074 | .016 | -.333 | -4.582 | <.001 | .530 | 1.887 |
|  | Honesty-Humility | .970 | .885 | .063 | 1.097 | .274 | .855 | 1.169 |
|  | Emotionality | .962 | .858 | .073 | 1.122 | .263 | .662 | 1.510 |
|  | Extraversion | .220 | .907 | .020 | .243 | .808 | .414 | 2.415 |
|  | Agreeableness | -.479 | .805 | -.035 | -.596 | .552 | .804 | 1.245 |
|  | Conscientiousness | -1.278 | .976 | -.079 | -1.310 | .191 | .774 | 1.293 |
|  | Openness | -1.279 | .700 | -.106 | -1.829 | .069 | .838 | 1.193 |

Dependent Variable: BMI


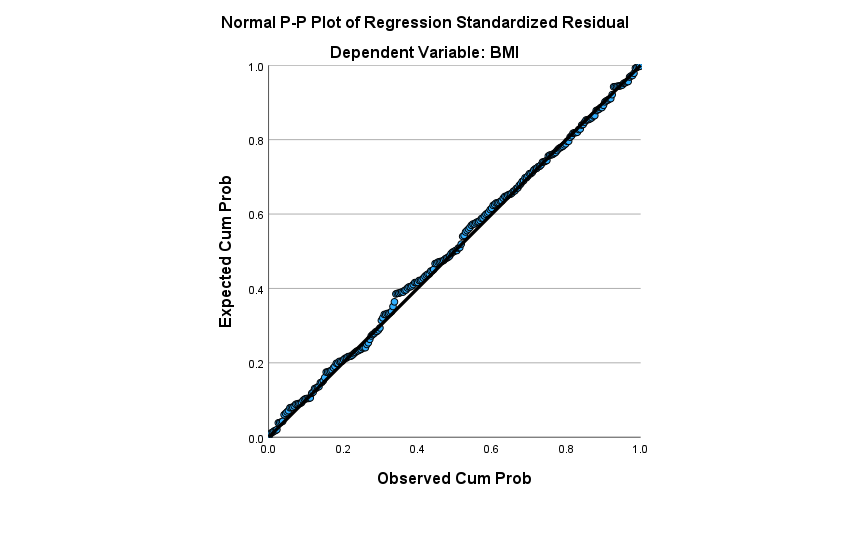

Supplement: S1 Data — (DOCX) [file pone.0292553.s006.docx]
